# A visible-light-driven composite photocatalyst of TiO_2_ nanotube arrays and graphene quantum dots

**DOI:** 10.3762/bjnano.5.81

**Published:** 2014-05-22

**Authors:** Donald K L Chan, Po Ling Cheung, Jimmy C Yu

**Affiliations:** 1 Department of Chemistry and Shenzhen Research Institute, The Chinese University of Hong Kong, Shatin, New Territories, Hong Kong, China

**Keywords:** anodic oxidation, graphene quantum dots, photocatalyst, photodegradation, TiO_2_ nanotube arrays

## Abstract

TiO_2_ nanotube arrays are well-known efficient UV-driven photocatalysts. The incorporation of graphene quantum dots could extend the photo-response of the nanotubes to the visible-light range. Graphene quantum dot-sensitized TiO_2_ nanotube arrays were synthesized by covalently coupling these two materials. The product was characterized by Fourier-transform infrared spectrometry (FTIR), scanning electron microscopy (SEM), transmission electron microscopy (TEM), X-ray diffraction (XRD), thermogravimetric analysis (TGA) and UV–vis absorption spectroscopy. The product exhibited high photocatalytic performance in the photodegradation of methylene blue and enhanced photocurrent under visible light irradiation.

## Introduction

Semiconductor-mediated photocatalysis is a promising technique for the conversion of solar energy as well as degradation of organic pollutants in air and water [1–2]. Among various photocatalysts, nanostructured titanium dioxide (TiO_2_) is the most widely used because of its high activity, long-term stability and low production cost [3–4]. However, pure TiO_2_ is not efficient for solar-driven applications because it requires UV excitation [5]. Belonging to one-dimensional nanostructures, TiO_2_ nanotube arrays (TNAs) synthesized by anodic oxidation of titanium had attracted particular interests [6–7]. Comparing with bulk nanoparticles, smooth walls of nanotubes provide a lower surface state density hence lowering recombination probability. Random walk of charges is suppressed because of the one-dimensional nature of the tubes [8]. Moreover, nanotube layers do have higher surface area for more active reaction sites over the bulk nanoparticle layers [9] and they were shown to be more efficient in photocatalysis [10]. Since TNAs can be grown directly on a conducting Ti substrate, they can be used directly as photoanodes for various applications. The activity of TNAs can be further enhanced by applying a potential bias [11]. In the recent years, TNAs have been widely studied for their applications in solar cells [12–14] or photoreactors [15–16]. Various approaches have been developed to achieve photoresponse of TiO_2_-based catalysts towards visible light, for example, doping with metal or non-metal [7,17–20], coupling with other semiconductor materials to form composite catalysts [4,21–24]. Two-dimensional graphene has attracted immense attention due to its large specific area, high intrinsic electron mobility and good electrical conductivity [3]. As an excellent electron acceptor, graphene has been combined with semiconductor photocatalysts such as TiO_2_ [25], ZnO [26] and CdS [27] to enhance their photocatalytic activities. However, graphene sheets are usually micrometer-sized and they can hardly be introduced into efficient nano-sized photocatalysts on a solid support, for example, TNAs.

Zero-dimensional graphene quantum dots (GQDs) are defined as few-layered graphene with lateral dimensions smaller than 100 nm [28]. Due to quantum confinement and edge effects, GQDs possess a size-dependent band gap and other interesting properties [29–30]. In recent years, GQDs have been explored for their potential applications in bioimaging [31], sensing [32], photovoltaics [33–34]. Besides, they have been coupled with TiO_2_ nanoparticles to achieve visible-light-driven photocatalysis [35–36]. Very recently, the combination of GQDs with CdS-modified TNAs was reported for photoelectrochemical hydrogen production. However, GQDs did not enhance the activity of bare TNAs in the study [37]. GQDs have also been chemically coupled with ZnO nanowires for photoelectrochemical water splitting [38].

**Scheme 1:**
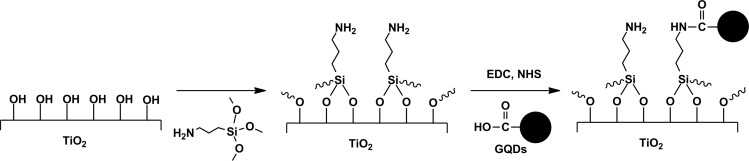
Reaction scheme for the loading of GQDs onto TNAs via covalent bonding.

In the present work, a composite photocatalyst of graphene quantum dots and TiO_2_ nanotube arrays (GQDs/TNAs) was fabricated by the coupling reaction between carboxyl-containing GQDs and amine-functionalized TNAs (Scheme 1). The experimental data revealed that sensitization of TNAs with GQDs not only extended the optical absorption spectrum of TNAs over the visible range, but also enhanced the photocatalytic and photoelectrochemical performances of TNAs under visible light.

## Results and Discussion

**Figure 1:**
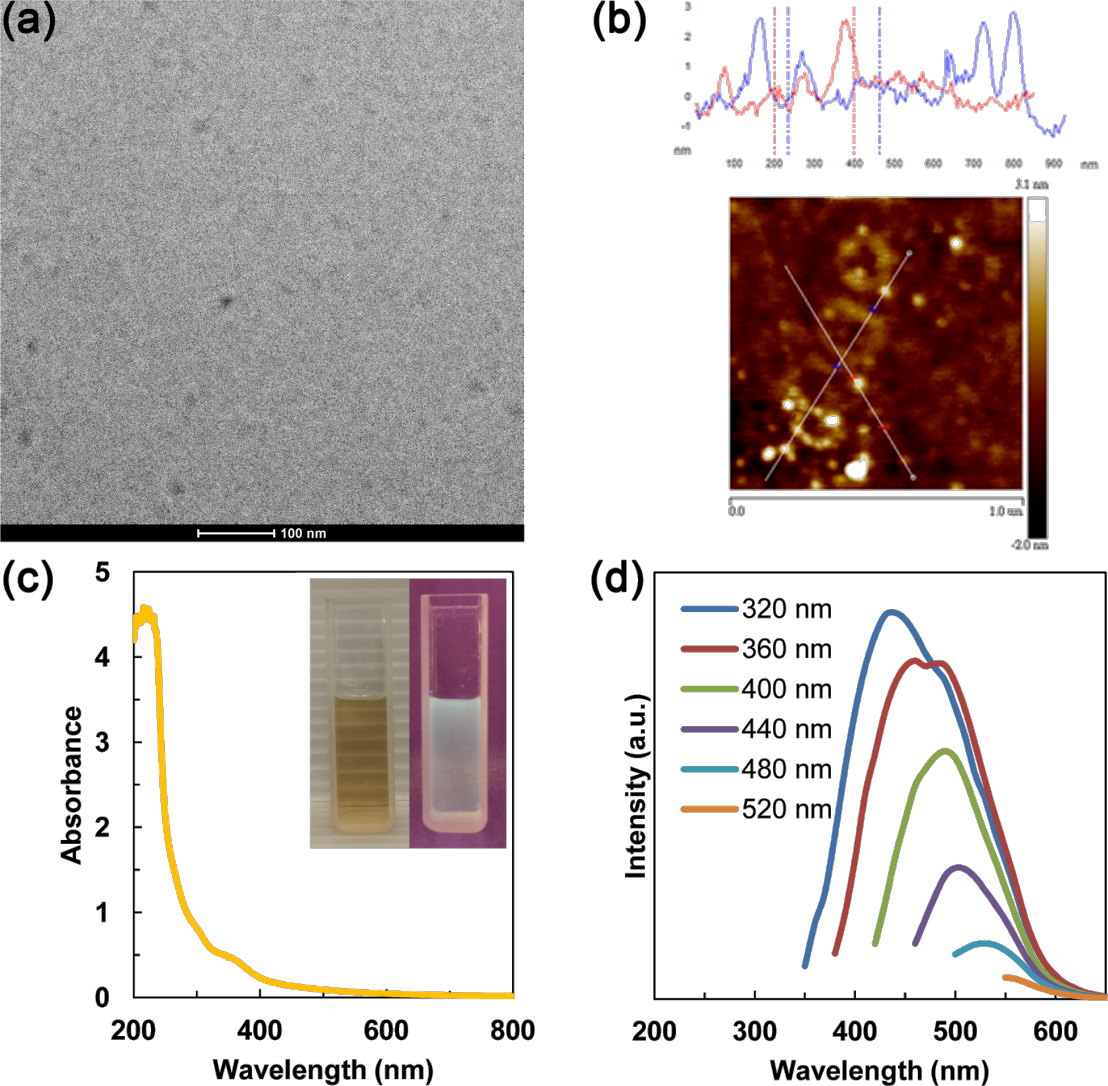
(a) TEM image of GQDs, (b) AFM image of GQDs with corresponding height profile, (c) UV–vis absorption spectrum of GQDs, inset: photos of GQDs in aqueous solution under ambient light (left) and 365 nm UV light (right), (d) PL spectra of GQDs at different excitation wavelengths.

Figure 1a shows a TEM image of GQDs with diameters of about 10 nm. The AFM image and a corresponding height profile in Figure 1b suggest that the thickness of the GQDs were between 0.5 and 3 nm, corresponding to one to few layers of graphene [39]. According to the UV–vis absorption spectrum in Figure 1c, GQDs show a broad absorption below 600 nm and a small peak at ca. 340 nm. The result agrees with typical absorption spectra of GQDs being reported [28]. Figure 1d shows the excitation-dependent emission of GQDs. This behavior could be explained by the differences in size and emissive states of GQDs [40]. To provide evidence for the existence of carboxyl groups in GQDs, Fourier-transform infrared (FTIR) spectra of GO and GQDs were obtained (Figure S1, Supporting Information File 1).

**Figure 2:**
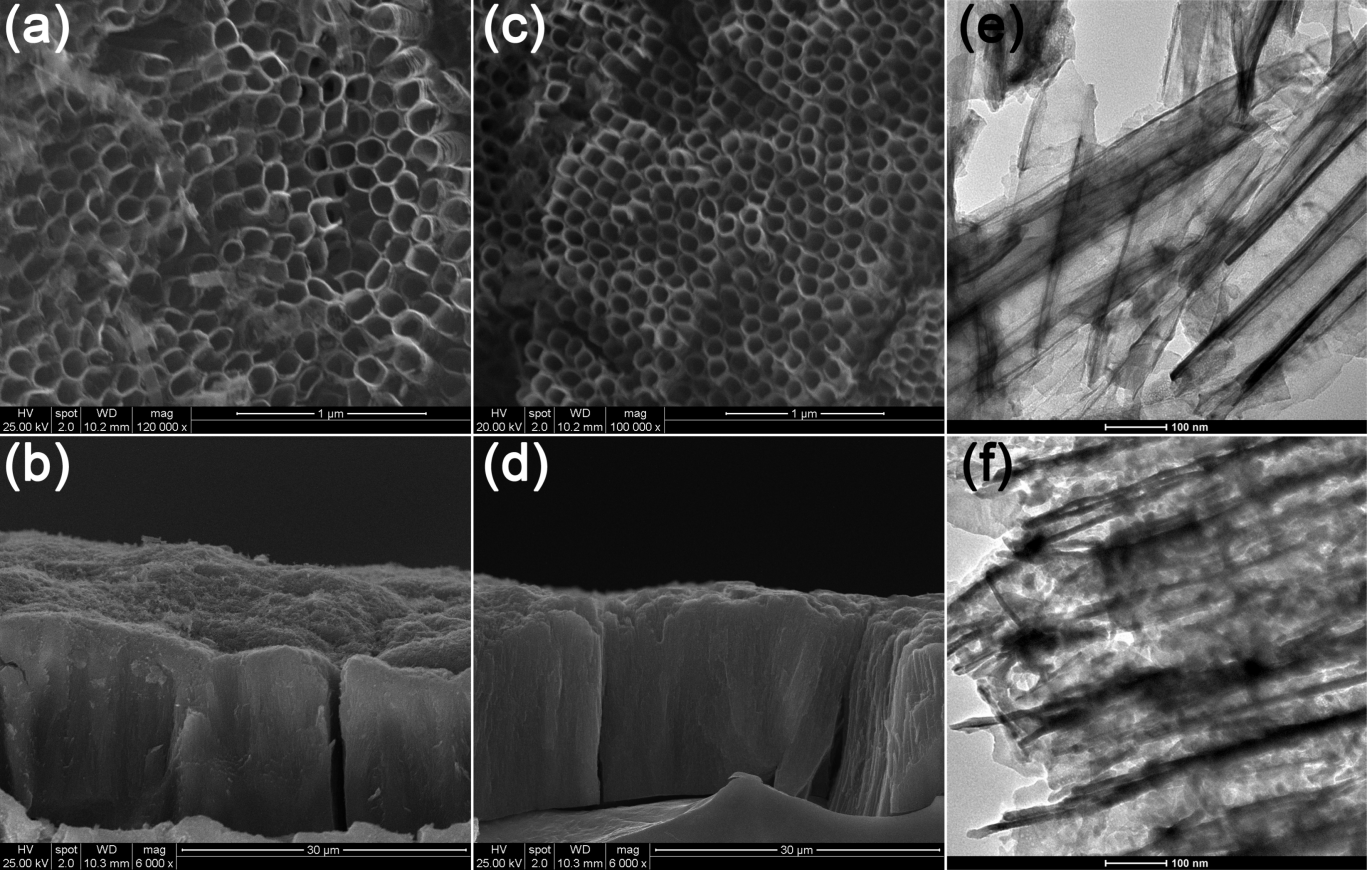
FESEM images of (a,b) pristine TNAs and (c,d) GQDs/TNAs; TEM images of (e) pristine TNAs and (f) GQDs/TNAs.

FESEM and TEM were used to examine the morphology of the TNAs. Figure 2 shows typical FESEM images with top (a, c) and side (b, d) views of the prepared TNAs films. The nanotube arrays are highly ordered and vertically aligned. Each nanotube has an inner diameter of approximately 110 nm and a length of about 18 μm. As shown in Figure 2c and Figure 2d, the TNAs retain the morphology after coupling with GQDs. In Figure 2e, the hollow structure of nanotubes can be observed clearly. In Figure 2f, dark spots with diameters of about 10 nm can be found, suggesting the successful loading of GQDs onto TNAs.

**Figure 3:**
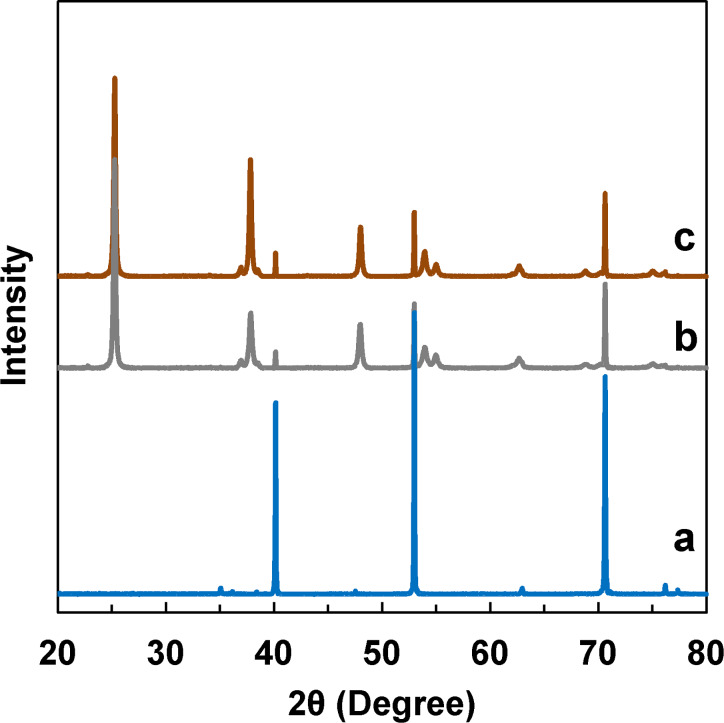
XRD patterns of (a) Ti foil, (b) TNAs and (c) GQDs/TNAs.

The structures of the products were investigated by using XRD. Figure 3 shows the XRD pattern of a pure Ti foil, which is consistent with the standard (JCPDS 44-1294). Figure 3 also shows that TNAs and GQDs/TNAs exhibit the same diffraction peaks at 2θ of 25.3°, 36.9°, 37.8°, 38.5°, 48.0°, 53.9°, 55.0°, 62.7°, 68.8° and 70.6°. These peaks match very well with anatase TiO_2_ (JCPDS 21-1272). FTIR spectra were also obtained to study the chemical structures of the products (Figure S2, Supporting Information File 1).

**Figure 4:**
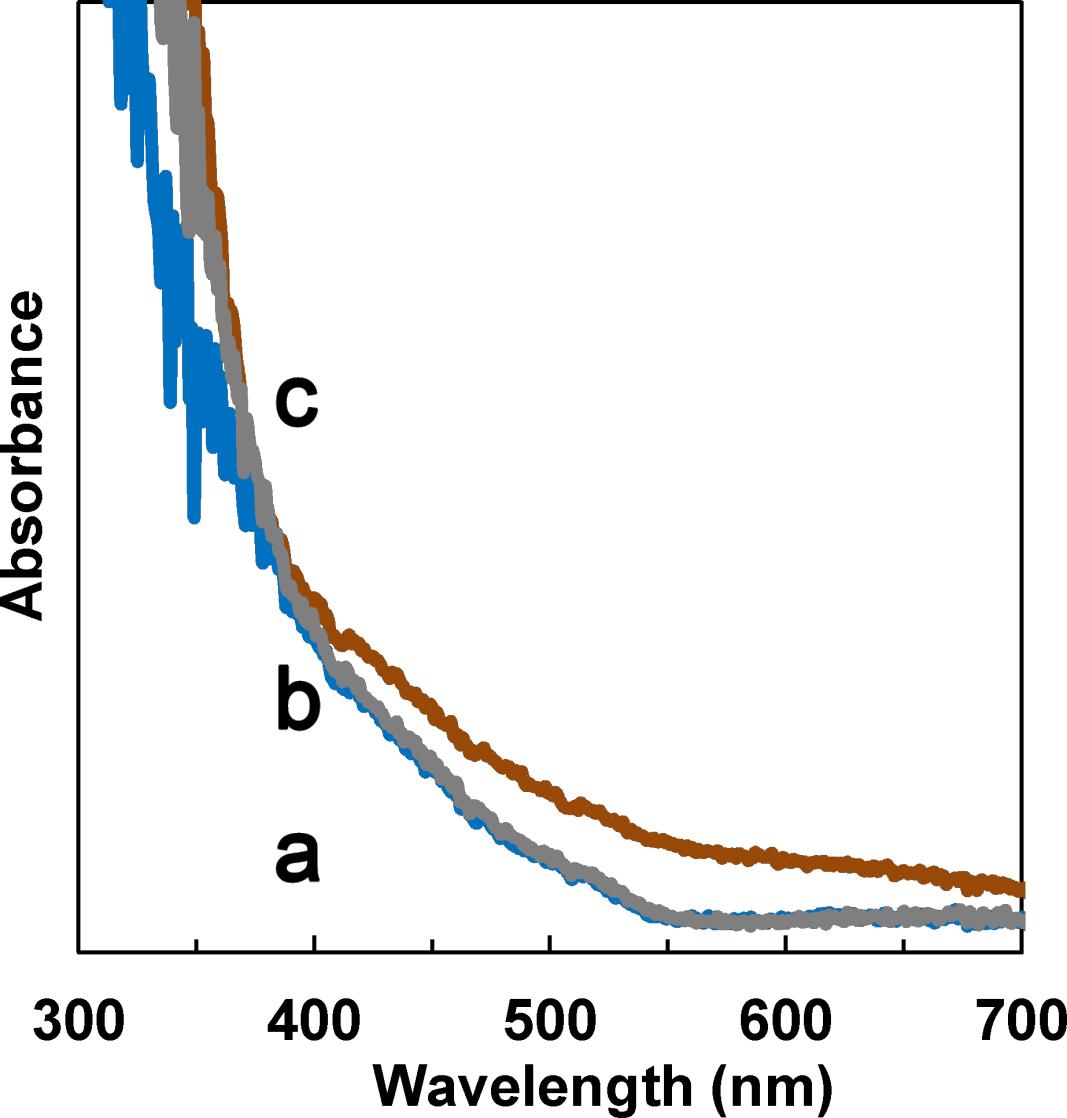
UV–vis absorption spectra of (a) TNAs, (b) amine-functionalized TNAs and (c) GQDs/TNAs.

Figure 4 shows the UV–vis absorption spectra of the samples. For pristine TNAs, the absorption edge extends up to about 550 nm instead of a typical value of TiO_2_ (400 nm). This phenomenon can be explained by the incorporation of nitrogen into the nanotubes from NH_4_F during anodization. A subsequent annealing at 450 °C resulted in the formation of N 2p states above the valence band of TiO_2_ and hence in a red shift of the absorption edge [41]. The absorption spectrum of amine-functionalized TNAs is similar to that of pristine TNAs. For GQDs/TNAs, higher absorption intensity at wavelengths from 400 to 600 nm is observed, indicating the visible light response of TNAs is enhanced by loading GQDs.

**Figure 5:**
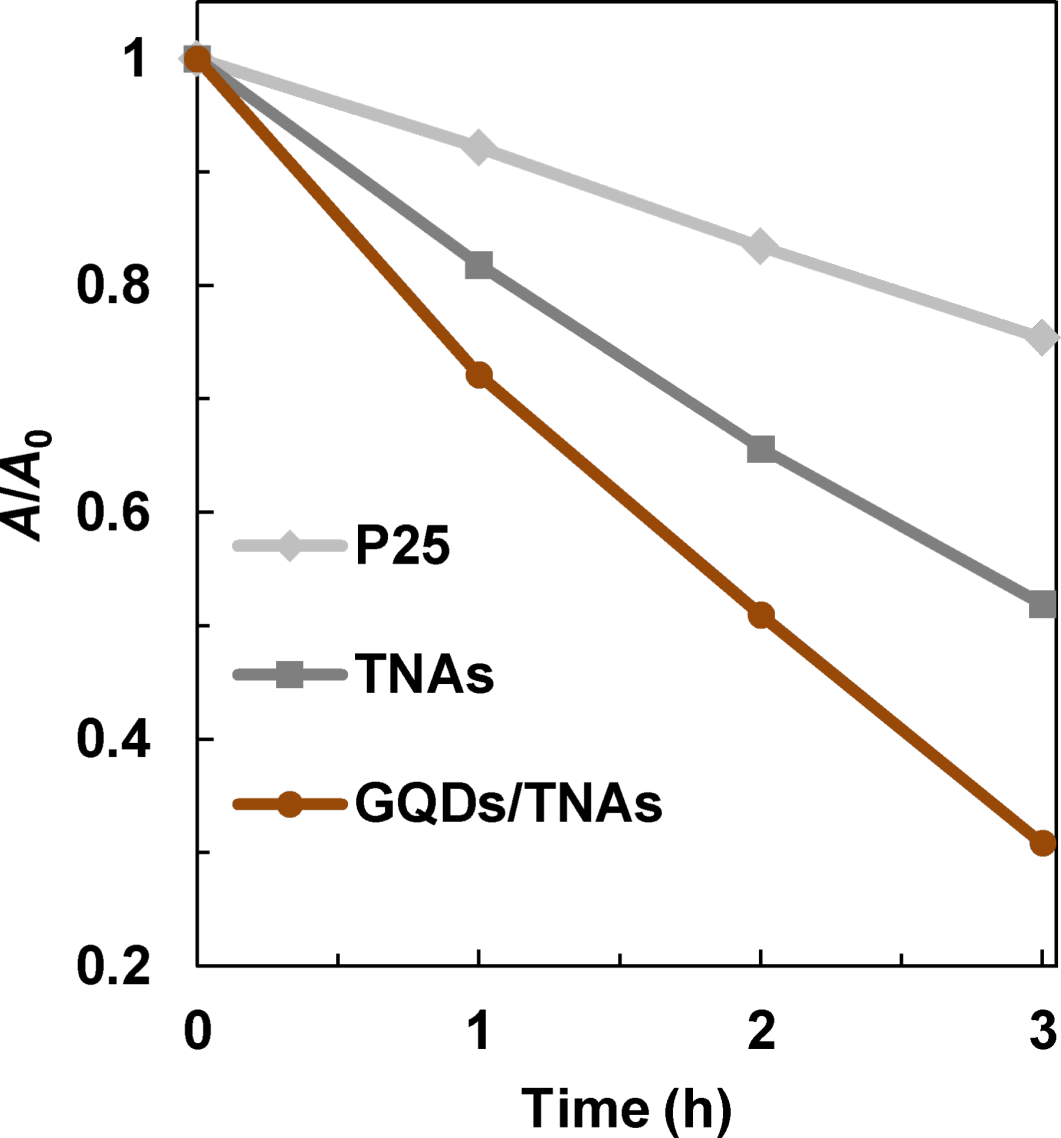
Photodegradation of methylene blue for TNAs and GQDs/TNAs under visible light irradiation.

The photocatalytic activities of the catalysts were evaluated by the degradation of MB under visible light irradiation. Figure 5 shows that pristine TNAs have a relatively low activity. The concentration of MB dropped slowly to about 52 % after 3 h irradiation. The activity of pristine TNAs under visible light can be explained by nitrogen-doping as described previously. For GQDs/TNAs, an enhanced activity is achieved. The concentration of MB dropped to about 31% after 3 h irradiation. For comparison, the activity of pure TiO_2_ (P25) was tested under the same conditions and it was found to be low.

**Figure 6:**
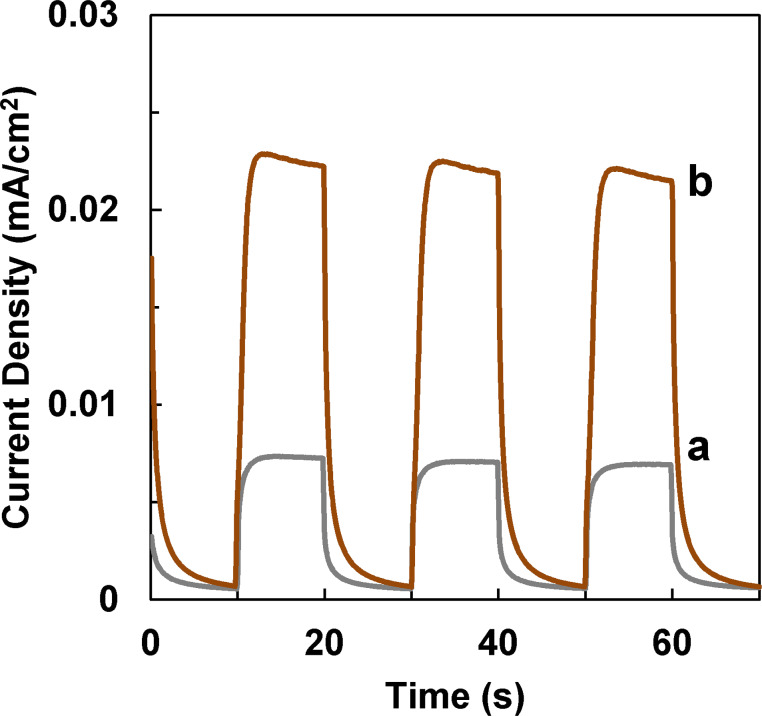
Photocurrent responses of (a) TNAs and (b) GQDs/TNAs under visible-light irradiation. The potential bias was 0.417 V.

TNAs were stably grown on a conducting Ti substrate, so the entire foil can be directly used for photoelectrochemical applications. Photocurrent responses of the catalysts were measured under visible light irradiation. Figure 6 clearly shows a significant enhancement of photocurrent after the loading of GQDs, indicating the charge separation efficiency of TNAs is greatly enhanced. The stable current reveals that GQDs are covalently bonded to TNAs instead of adsorbed onto the surface of TNAs.

The improved photocatalytic performance of GQDs/TNAs over TNAs can be simply explained by the photosensitization of TNAs by GQDs [38]. Upon visible light irradiation, electron–hole pairs are generated by the GQDs. Typically, the conduction band level of GQDs is higher than that of TiO_2_ [36,42]. Thus, an interfacial electron transfer from GQDs to TNAs is possible. Meanwhile, such a directional charge transfer promotes charge separation and reduces the probability of charge recombination, then further increases the activity of the photocatalyst.

## Conclusion

In summary, a visible-light-driven photocatalyst was fabricated by covalently bonding GQDs onto amine-modified TNAs. The GQDs/TNAs composite retains the highly ordered nanotube morphology and well crystallized anatase phase. The high visible-light photocatalytic activity could be attributed to photosensitization of TNAs by GQDs. This research shows the potential of GQD-based photocatalysts for visible-light-driven photocatalytic and photoelectrochemical applications.

## Experimental

**Synthesis of TiO_2_ nanotube arrays (TNAs):** Highly ordered TNAs were fabricated by an anodic oxidation approach [43]. Ethylene glycol (99+%) containing 0.5 wt % NH_4_F and 2.0 wt % deionized (DI) water was used as electrolyte. Ti foil (2 cm × 3 cm) was used as a working electrode, and a Pt foil (1 cm × 1 cm) served as a counter electrode. Prior to anodization, Ti foils were washed with ethanol, acetone by ultrasonication to remove contaminants, subsequently rinsed with DI water and dried in air. At room temperature, anodization is carried out by immersing a Ti foil in as-prepared electrolyte for 3 h at 60 V. Afterwards, the sample was removed from the electrochemical cell, rinsed with DI water, sonicated in ethanol for 2 min to remove surface debris. A subsequent heating to 450 °C for 1 h with a temperature increasing rate of 1 °C·min^−1^ in air was applied to improve crystallization.

**Synthesis of graphene quantum dots (GQDs):** GQDs were synthesized from graphene oxide (GO) by heating with a solution of hydrogen peroxide and ammonia [44]. 20 mg of GO was dispersed into 5 mL of water and sonicated for 10 minutes. 40 mL of H_2_O_2_ (30%) and 10 mL of NH_3_ (28–30%) were added to the dispersion. The mixture was then stirred at 80 °C for 24 h followed by centrifugation for 10 minutes to remove large GO. The supernatant was heated at 60 °C under reduced pressure to remove H_2_O_2_, NH_3_ and water. The solid GQDs were re-dispersed into water for further use.

**Synthesis of GQDs/TNAs:** TNAs were firstly immersed in 0.2 wt % (3-aminopropyl)trimethoxysilane (APTMS) in toluene for 3 h, rinsed with toluene and dried. The modified TNAs were then immersed in a beaker containing a solution of GQDs (1 mg·mL^−1^), ethyl(dimethylaminopropyl)carbodiimide (EDC) and *N*-hydroxysuccinimide (NHS) for 4 h. The foils were then sonicated in DI water, rinsed with DI water and dried in air.

**Characterization:** The morphologies of the products were characterized by transmission electron microscopy (Philips, CM120) and field-emission scanning electron microscopy (FEI, Quanta 400 FEG). AFM images were obtained using a tapping mode with an atomic force microscope (Bruker, Dimension Icon). UV–vis spectra were recorded on a UV–vis spectrometer (Varian, Cary 100). The PL measurements were performed using a fluorescence spectrometer (Hitachi, F-4500). X-ray diffraction (XRD) patterns were recorded using a diffractometer (Bruker, D8 Advance) with high-intensity Cu Kα1 irradiation (λ = 1.5406 Å). The chemical structures of the products were characterized using a Fourier-transform infrared spectrometer (Nicolet, Magna 560). Thermogravimetric analysis was performed in air using a thermogravimetric analyzer (Perkin Elmer, TGA 6). The samples were heated from 50 °C to 800 °C at a rate of 10 °C·min^−1^.

**Photocatalytic activity measurements:** The photocatalytic activities of catalysts were evaluated by measuring the photodegradation of methylene blue (MB). In a typical measurement, 10 mg photocatalyst were mechanically detached from Ti foils and suspended in 20 mL of 10 ppm aqueous solution of MB. The solution was stirred in the dark for 12 h to reach the adsorption/desorption equilibrium. The suspension was then illuminated with a 300 W tungsten halogen lamp with a 400 nm cutoff filter. Photodegradation of MB was monitored by measuring the UV–vis absorption of the suspensions at regular time intervals. The suspension was centrifuged for 2 min to remove the photocatalyst before measurement. The peak absorbance of MB at 664 nm was used to determine its concentration.

**Photocurrent response measurements:** The photo-electrochemical measurements were performed in a three-electrode electrochemical cell by using a CHI 660D electrochemical workstation. The as-prepared TNAs, Pt foil (1.0 cm × 1.0 cm) and Ag/AgCl were the working, counter and reference electrodes. The electrolyte was a 0.1 M Na_2_SO_4_ aqueous solution. A 300 W xenon arc lamp was used as the irradiation source and the average light intensity was about 100 mW·cm^−2^. The photocurrent responses under illumination of visible light (AM 1.5G plus a 400 nm cutoff filter) were analyzed.

## Supporting Information

Supporting information features FTIR spectra, TGA profiles of the samples and UV–vis absorption spectra of methylene blue.

File 1 Additional figures.
