# Supplementary material for: A visible-light-driven composite photocatalyst of TiO2 nanotube arrays and graphene quantum dots
Source: Beilstein J Nanotechnol. 2014 May 22;5:689–95. doi: 10.3762/bjnano.5.81 (PMC4077368; doi:10.3762/bjnano.5.81)
Supplement: File 1 — Additional figures. [file Beilstein_J_Nanotechnol-05-689-s001.pdf]

Supporting Information

for

A visible-light-driven composite photocatalyst of TiO_2 nanotube arrays and graphene quantum dots

Donald K. L. Chan, Po Ling Cheung and Jimmy C. Yu*

Address: Department of Chemistry and Shenzhen Research Institute, The Chinese University of Hong Kong, Shatin, New Territories, Hong Kong, China

Email: Jimmy C. Yu* - jimyu@cuhk.edu.hk

* Corresponding author

Additional figures

Fourier-transform infrared (FTIR) analysis

GQDs could be loaded on TNAs by the formation of amide linkages. Both carboxyl groups in GQDs and amine groups in TNAs are essential in the reaction. To provide evidence for the existence of carboxyl groups in GQDs, Fourier-transform infrared (FTIR) spectra of GO and GQDs were obtained. Major peaks were assigned in Figure S1. For GO, skeletal vibration of graphite domains (ca. 1640 cm^{-1}) and C=O stretching in carboxylic acid moieties (ca. 1730 cm^{-1}) can be clearly observed. Similar peaks can be observed in the FTIR spectrum of GQDs. Nitrogen-related bonds can also be found in the spectrum, revealing the nitrogen-containing nature of GQDs prepared by using ammonia [1,2].

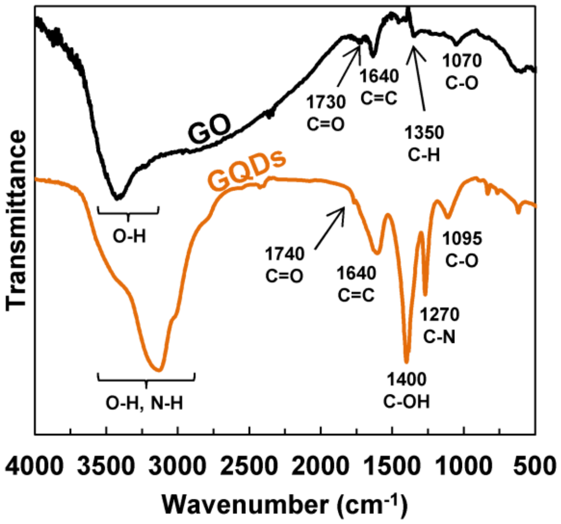

Figure S1: FTIR spectra of GO and GQDs.

In Figure S2, amine-functionalized TNAs show characteristic peaks corresponding to Si–O–Si and Ti–O–Si bonds [3], thus confirming the functionalization of TNAs. For GQDs/TNAs, the new peak at 1240 cm^{-1} can be attributed to C–N stretching vibrations from amide groups, indicating the formation of covalent linkages between GQDs and TNAs.

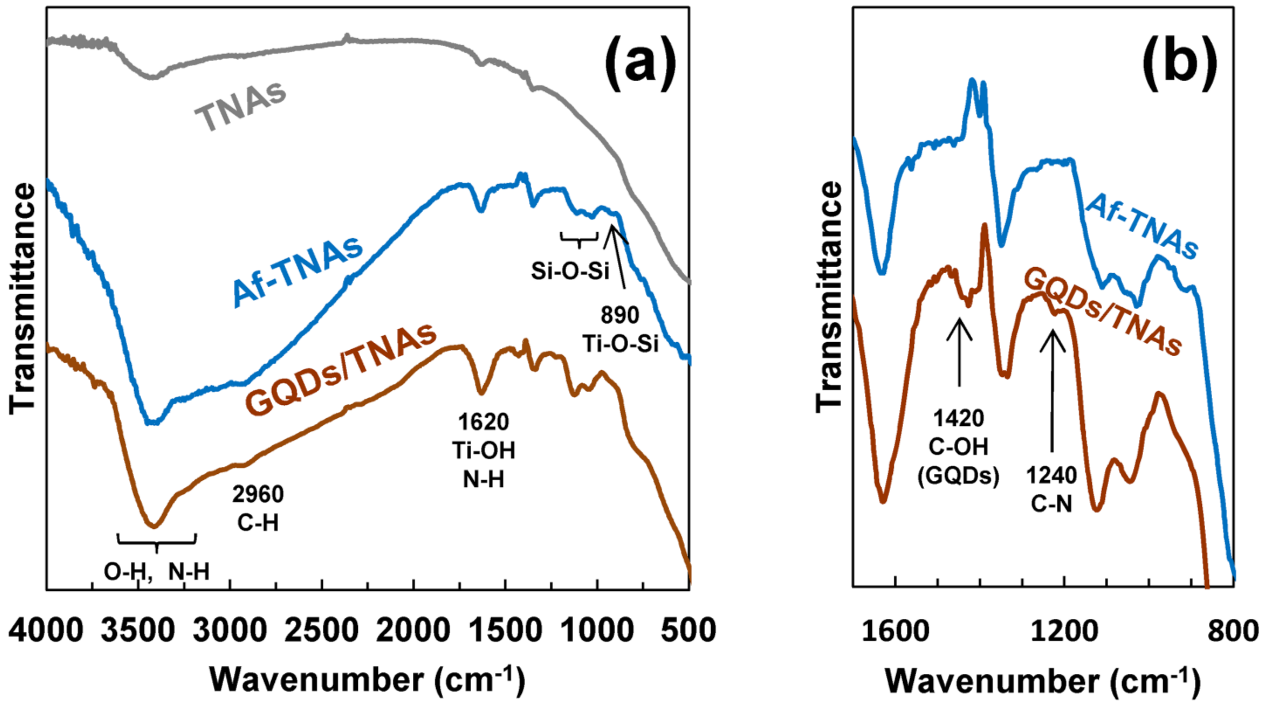

Figure S2: (a) FTIR spectra of TNAs, amine-functionalized TNAs (Af-TNAs) and GQDs/TNAs. (b) FTIR spectra of Af-TNAs and GQDs/TNAs ranged from 800 cm^{-1} to 1700 cm^{-1}.

Thermogravimetric analysis (TGA)

TGA measurements were carried out to estimate the quantity of GQDs in the composite photocatalyst. From Figure S3, amine-functionalized TNAs and

GQDs/TNAs show similar weight loss from 50 to 200 °C, which can be attributed to the loss of water adsorbed on the surface of TNAs. Starting from 250 °C, the weight loss is mainly due to (i) decomposition of aminosilane conjugated with TNAs, (ii) decomposition of nitrogen and oxygen containing groups on GQDs and (iii) decomposition of core parts of GQDs. By subtracting the weight losses of the two samples, the weight ratio of GQDs in the composite is estimated to be 1.9%.

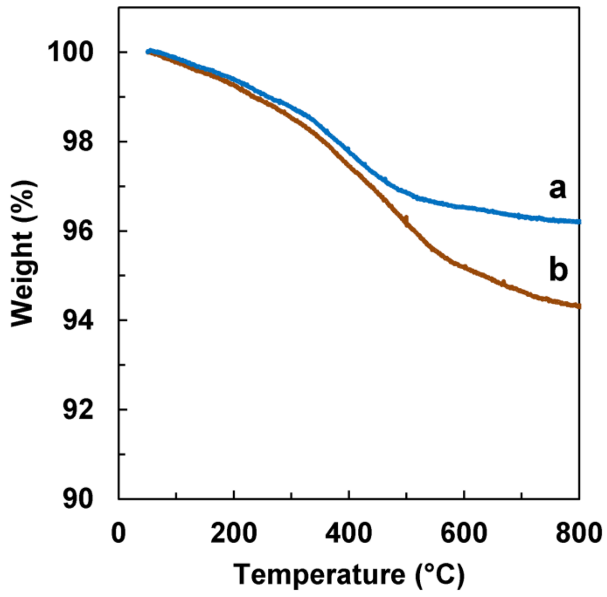

Figure S3: TGA profiles of (a) amine–functionalized TNAs and (b) GQDs/TNAs.

Spectral changes of methylene blue during photocatalytic degradation

The photodegradation of methylene blue was carried out for three cycles to investigate the stability of GQDs/TNAs. As shown in Figure S4a, there is no significant decrease in photodegradation rate during the three reaction cycles, indicating the good stability of the photocatalyst.

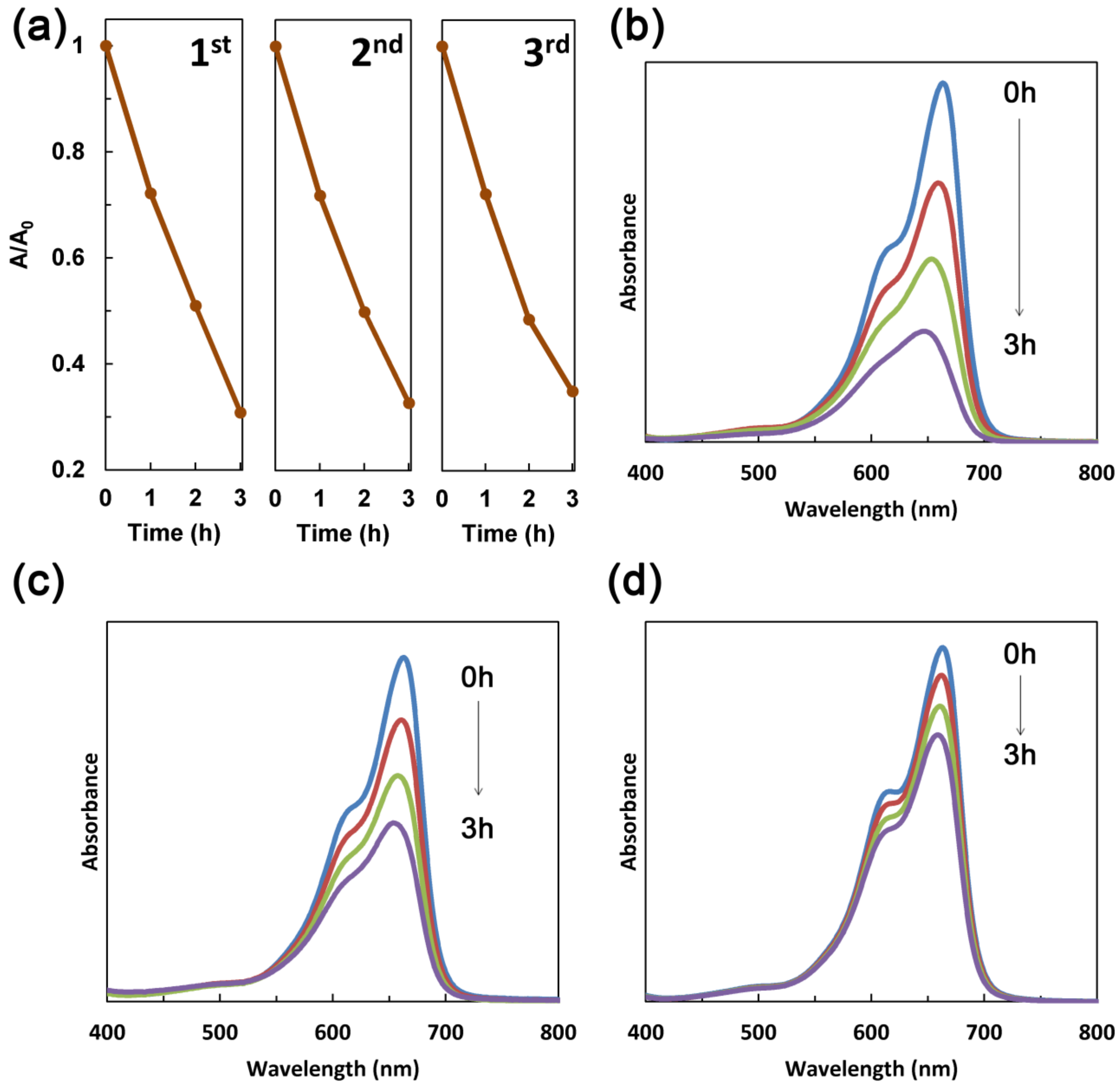

Figure S4: (a) Repeating photodegradation of methylene blue for GQDs/TNAs, (b–d) Time-dependent UV–vis absorption spectra of methylene blue under visible light. (b) GQDs/TNAs, (c) TNAs, (d) P25.

The amount of graphene was reported as an important factor for the photocatalytic activity of graphene-based composites [4,5]. Figure S5 shows the activity of TNAs

with different GQDs loading. 0.5 wt % GQDs/TNAs was prepared by reducing the duration of coupling reaction from 4 h to 1 h.

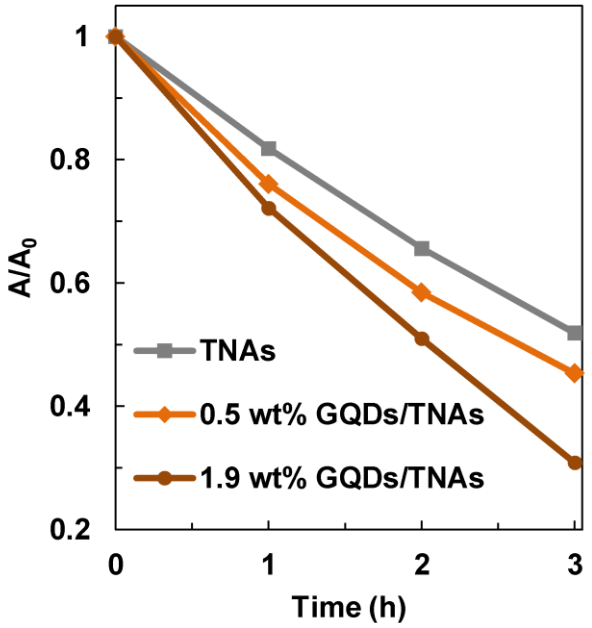

Figure S5: Photodegradation of methylene blue for TNAs with different weight ratios of GQDs under visible light irradiation.

References

1. Li, Y.; Zhao, Y.; Cheng, H.; Hu, Y.; Shi, G.; Dai, L.; Qu, L. *J. Am. Chem. Soc.* **2012,** *134*, 15–18. doi:10.1021/ja206030c
2. Jiang, F.; Chen, D.; Li, R.; Wang, Y.; Zhang, G.; Li, S.; Zheng, J.; Huang, N.; Gu, Y.; Wang, C.; Shu, C. *Nanoscale* **2013,** *5*, 1137–1142. doi:10.1039/c2nr33191h

3. Ye, L.; Pelton, R.; Brook, M. A. *Langmuir* **2007,** *23,* 5630–5637. doi:10.1021/la0626656

4. Jia, L.; Wang, D.; Huang, Y.; Xu, A.; Yu, H. *J. Phys. Chem. C* **2011,** *115,* 11466–11473. doi:10.1021/jp2023617

5. An, X.; Yu, J. C.; Wang, Y.; Hu, Y.; Yu, X.; Zhang, G. *J. Mater. Chem.* **2012,** *22,* 8525–8531. doi:10.1039/c2jm16709c
